# Spray-Dried Powder Formulation of Capreomycin Designed for Inhaled Tuberculosis Therapy

**DOI:** 10.3390/pharmaceutics13122044

**Published:** 2021-11-30

**Authors:** Zitong Shao, Waiting Tai, Yingshan Qiu, Rico C. H. Man, Qiuying Liao, Michael Y. T. Chow, Philip C. L. Kwok, Jenny K. W. Lam

**Affiliations:** 1 Department of Pharmacology and Pharmacy, Li Ka Shing Faculty of Medicine, The University of Hong Kong, 21 Sassoon Road, Pokfulam, Hong Kong, China; u3006730@connect.hku.hk (Z.S.); u3004556@connect.hku.hk (Y.Q.); u3542606@connect.hku.hk (R.C.H.M.); liaoqy@connect.hku.hk (Q.L.); 2 Advanced Drug Delivery Group, Sydney Pharmacy School, Faculty of Medicine and Health, The University of Sydney, Sydney, NSW 2006, Australia; wtai6746@uni.sydney.edu.au (W.T.); yee.chow@sydney.edu.au (M.Y.T.C.); philip.kwok@sydney.edu.au (P.C.L.K.); 3 Advanced Biomedical Instrumentation Centre, Hong Kong Science Park, Shatin, New Territories, Hong Kong, China

**Keywords:** capreomycin, dry powder aerosol, inhalation, pulmonary delivery, spray drying, tuberculosis

## Abstract

Multi-drug-resistant tuberculosis (MDR-TB) is a huge public health problem. The treatment regimen of MDR-TB requires prolonged chemotherapy with multiple drugs including second-line anti-TB agents associated with severe adverse effects. Capreomycin, a polypeptide antibiotic, is the first choice of second-line anti-TB drugs in MDR-TB therapy. It requires repeated intramuscular or intravenous administration five times per week. Pulmonary drug delivery is non-invasive with the advantages of local targeting and reduced risk of systemic toxicity. In this study, inhaled dry powder formulation of capreomycin targeting the lung was developed using spray drying technique. Among the 16 formulations designed, the one containing 25% capreomycin (*w*/*w*) and spray-dried at an inlet temperature of 90 °C showed the best overall performance with the mass median aerodynamic diameter (MMAD) of 3.38 μm and a fine particle fraction (FPF) of around 65%. In the pharmacokinetic study in mice, drug concentration in the lungs was approximately 8-fold higher than the minimum inhibitory concentration (MIC) (1.25 to 2.5 µg/mL) for at least 24 h following intratracheal administration (20 mg/kg). Compared to intravenous injection, inhaled capreomycin showed significantly higher area under the curve, slower clearance and longer mean residence time in both the lungs and plasma.

## 1. Introduction

Tuberculosis (TB) is an airborne communicable disease caused by *Mycobacterium tuberculosis* (*Mtb*). It is one of the major causes of illness and death worldwide. As an unresolved public health threat, it is estimated that 10 million people fell ill with TB in 2019 [1]. Although the number of infections has slowly declined in recent years, the coronavirus disease 2019 (COVID-19) pandemic and the increasing number of human immunodeficiency virus (HIV) infections exert negative impact on global TB control [1]. The management of TB has become more challenging with the emergence of multi-drug-resistant TB (MDR-TB), which refers to the resistance to both isoniazid and rifampicin. Treatment of MDR-TB requires the use of second-line drugs that are mostly injectables with high toxicity and long treatment duration of at least nine months (typically up to 24 months) [1,2].

Capreomycin is a cyclic polypeptide antibiotic composed of four molecular analogues (IA, IB, IIA and IIB) isolated from *Streptomyces capreolus* [3]. It is a second-line anti-TB agent used for the treatment of MDR-TB. The mechanism of action of capreomycin is not completely clear but it is widely accepted that it inhibits bacterial protein synthesis by binding to the 70S ribosomal unit. It can interfere with several ribosomal functions, including the formation of the 30S subunit initiation complex and the translocation of tRNA [4,5]. Capreomycin needs to be repeatedly injected (up to five times per week) and is associated with severe adverse effects such as nephrotoxicity and ototoxicity. Therefore, long-term administration of capreomycin is a heavy load for patients, especially the elderly and those with renal impairment [6].

Pulmonary delivery is a non-invasive alternative route of drug administration for the treatment of pulmonary TB, including MDR-TB. By delivering the drug to the lung directly, high local drug concentration at the site of primary infection can be achieved. As a result, a lower dose is required, thereby minimising systemic exposure, improving drug efficacy and reducing the risk of drug resistance development [7]. Another advantage of inhaled TB therapy is that particles targeted to the lung can be phagocytosed by alveolar macrophages in which the *Mtb* colonized [8]. Compared to liquid formulations, dry powder formulations have better stability, which is convenient for storage and transportation. In addition, dry powder inhalers (DPIs) are easy to operate and allow the delivery of high doses, which are often required for antibiotics [9]. A few studies have reported the preparation of inhalable powder of capreomycin, some of which were formulated in liposomes or poly(lactic-*co*-glycolic acid) (PLGA) microparticles to control the particles size distribution for efficient lung deposition [10,11]. Hickey et al. produced spray-dried powder of capreomycin (as sulfate), in which 50% ethanol (*v*/*v*) was used as the solvent and l-leucine as dispersion enhancer [2,12,13,14]. Schoubben et al. blended spray-dried capreomycin (as sulfate) powders with lactose at a 1:50 ratio (*w*/*w*) to improve powder dispersibility [15]. Since TB therapy requires high dose of drug, it may not be practical to deliver capreomycin with such a large amount of excipient [16].

The aim of this study was to develop inhaled powder formulations of capreomycin that exhibit high drug loading, excellent aerosol performance and good pharmacokinetic profile compared to other previously reported formulations, in a method that is easy for scale-up without the use of organic solvent [2,8,10,11,12,13,14,15,17]. Mannitol was used in the present study as a bulking excipient to produce spray-dried powder of capreomycin for inhalation. It is frequently exploited as an excipient in spray-dried formulations with satisfactory aerosol performance and physical stability [9,18,19]. Formulations containing different drug contents were prepared by spray drying at different inlet temperatures in order to explore the effect on particle properties. The morphology, particle size distribution, aerosol performance, crystallinity and surface composition of the powder formulations were investigated. The optimal formulation was also identified to evaluate its pharmacokinetic profile in mice.

## 2. Materials and Methods

### 2.1. Materials

Capreomycin (Capastat^®^ sulfate) was purchased from Yick-Vic Chemicals and Pharmaceuticals (Hong Kong, China). Mannitol (Pearlitol^®^ 160) was purchased from Roquette (Lestrem, France). Analytical standard of capreomycin sulfate and trifluoroacetic acid (TFA) were obtained from Sigma-Aldrich (Poole, UK). Heptafluorobutyric acid (HFBA) of HPLC grade was obtained from Thermo Scientific Pierce (Rockford, IL, USA). All solvents and reagents were of analytical grade.

### 2.2. Preparation of Spray-Dried Powders

Capreomycin and mannitol were dissolved in 15 mL of ultra-pure water at four different drug: mannitol mass ratios of 1:4, 1:3, 1:2 and 1:1 to obtain a final solute concentration of 1% (*w*/*v*). Each formulation was spray-dried using a laboratory spray dryer (Büchi B-290, Labortechnik AG, Flawil, Switzerland) with the two-fluid nozzle (Büchi, with an internal diameter of 0.7 mm) at four different inlet temperatures of 60, 90, 120 and 150 °C. The nitrogen atomization flow rate was 742 L/h, the aspiration rate was 38 m³/min (100%) and the liquid feed rate was 2.1 mL/min. A total of 16 formulations were prepared (Table 1). The spray-dried powders were stored in a desiccator with silica gel at room temperature until further analysis. The production yield was defined as the mass of powder collected after spray drying divided by the total feed solid mass.

### 2.3. Drug Content

The drug content was determined as the measured amount of capreomycin with respect to the mass of the spray-dried powders. For each formulation, 1 mg of powder was weighed and dissolved in 5 mL of ultrapure water using a volumetric flask. The samples were filtered with 0.45 µm membrane filter (Nylon syringe filter, Membrane Solutions, Auburn, WA, USA) and quantified by high-performance liquid chromatography (HPLC) (Agilent 1260 Infinity; Agilent Technologies, Santa Clara, CA, USA). The HPLC method was adopted and modified according to a previous study [8]. In brief, a C18 column (5 μm, 4.6 × 250 mm, Agilent, Santa Clara, CA, USA) was used. The mobile phase was composed of 0.1% TFA aqueous solution (pH 2) and acetonitrile (95:5, *v*/*v*). The running flow rate was 1 mL/min. A volume of 50 μL sample was injected and capreomycin was detected by UV absorbance at 268 nm. Capreomycin IIA/IIB eluted at approximately 4.6 min while capreomycin IA/IB eluted at around 5.4 min. Total capreomycin was quantified by using a calibration curve with linearity (*R*^2^ = 0.9999) between 3.0 μg/mL and 400.0 μg/mL.

### 2.4. Morphology Study

Scanning electron microscopy (SEM) (Hitachi S-4800, Hitachi High-technologies Crop., Tokyo, Japan) was used to study the morphology of the spray-dried powders. Samples were sputter-coated with gold-palladium alloy (approximately 11 nm) after being dispersed on the adhesive carbon discs. The coated sample was imaged at 5.0 kV.

### 2.5. Particle Size Distribution

The volumetric size distribution of the spray-dried powders was measured by laser diffraction (HELOS/KR incorporated with an inhaler module, Sympatec, Clausthal-Zellerfeld, Germany) as previously reported [20]. Approximately 5 mg of powder was loaded in gelatin capsules and dispersed from a Breezhaler^®^ at a flow rate of 60 L/min and a pressure drop of 1.5 kPa. The 100 mm (R3) lens (measuring range 0.45–175 μm) was employed in the measurement. Each powder formulation was measured in triplicate. The tenth (D10), median (D50), and ninetieth (D90) percentile of the volumetric diameter were recorded. The span was calculated according to the formula (D90–D10)/D50.

### 2.6. In Vitro Aerosol Performance

The aerosol performance of the spray-dried powder was evaluated with a Next Generation Impactor (NGI) (Copley Scientific Limited, Nottingham, UK) without a pre-separator. For each formulation, a Size 3 gelatin capsule (Capsugel, Lonza, NSW, Australia) containing around 7 mg of spray-dried powders were placed in a Breezhaler^®^. The powders were dispersed at an airflow rate of 90 L/min for 2.7 s with a pressure drop of 3.5 kPa [20]. Before dispersion, the NGI plates were coated with the silicon lubricant (LPS Laboratories, Tucker, GA, USA). After dispersion, a volume of 4 mL ultrapure water was used to rinse and dissolve the powder in the capsule, inhaler, adaptor and each NGI plate separately. After filtering through a 0.45 µm membrane filter (Nylon syringe filter, Membrane Solutions, Auburn, WA, USA), the samples were assayed by HPLC as described above. The NGI experiments were performed in triplicate for each formulation. The recovered dose was defined as the total amount of powder in the capsule, inhaler, adaptor and all NGI stages assayed by HPLC in a single run. The emitted fraction (EF) referred to the fraction of the powder emitted from the inhaler with respect to the recovered dose. The fine particle dose (FPD) referred to the amount of powder with an aerodynamic diameter less than 5.0 μm, which was calculated by interpolation. Fine particle fraction (FPF) was the fraction of the fine particle dose with respect to the recovered dose. The mass median aerodynamic diameter (MMAD) was defined as the aerodynamic diameter at which 50% of the particles (collected from stage 1 to the micro-orifice collector) by mass are larger and 50% are smaller. The MMAD and geometric standard deviation (GSD) were calculated by a linear fit of the cumulative mass and aerodynamic cut-off diameter on a log scale.

### 2.7. Thermogravimetric Analysis (TGA)

Thermogravimetric analysis (TGA) was conducted to measure the residual moisture of the spray-dried powders. About 2 mg of each formulation was weighed into a 70 µL alumina crucible and heated from 25 to 150 °C at 10 °C/min with a 20 mL/min nitrogen flow in a TGA/DSC 1 STARe System (Mettler Toledo, Greifensee, Switzerland). The loss in mass indicated the residual moisture evaporated from the spray-dried powders.

### 2.8. Differential Scanning Calorimetry (DSC)

The thermal response profiles of each spray-dried powder formulation and raw materials were assessed by differential scanning calorimetry (DSC) (Model Q1000, TA Instruments, New Castle, DE, USA) as previously reported [21]. Between 1–3 mg of powder was loaded into an aluminum crucible, crimped with a non-perforated lid and heated from 20 °C to 200 °C at 10 °C /min under a 50 mL/min nitrogen purge.

### 2.9. Powder X-ray Diffraction (PXRD)

The crystalline structures of the spray-dried powders and raw materials were studied by X-ray powder diffraction (X’Pert Powder; Malvern Panalytical Ltd., Malvern, UK) as previously reported [20]. About 4 mg of powder was spread compactly on the sample plate and subjected to Cu-Kα radiation at a current of 45 mA and voltage of 40 kV under ambient temperature. The scattered X-rays were measured from 5 to 35° by a detector with a scan speed of 0.02° per second and a step size of 0.01°.

### 2.10. X-ray Photoelectron Spectroscopy (XPS)

The surface composition of the powders was examined by X-ray photoelectron spectroscopy (XPS) using a K-Alpha XPS system (Thermo Fisher Scientific, Waltham, MA, USA). Each sample was loaded onto carbon tape on the powder sample holder, followed by the measurement of the surface atomic concentration of carbon, oxygen, nitrogen, and sulfur. Hydrogen could not be detected by XPS so it was not measured. A monochromatic Al Kα X-ray source was operated at 72 W with a spot size of 400 µm to obtain the spectra. The pass energy of the survey and region scans were 200 eV and 50 eV, respectively. Similar procedures and settings for XPS have been adopted in other studies [22,23,24]. During measurement, the pressure in the analysis chamber was about 2 × 10^−7^ mbar and the charging of the samples was compensated by an electron flood gun. Six spots on each sample were randomly selected for measurement. Since capreomycin was the only component in the spray-dried samples containing nitrogen, its surface coverage was determined by calculating the ratio of nitrogen atoms on the surface of the spray-dried particles to those on raw capreomycin. The rest of the surface of the spray-dried powders was covered by mannitol. The expected atomic percentage of nitrogen was calculated using the following equation by assuming a homogenous distribution of the components of the particle according to the theoretical ratio of the components in the formulation. (The molecular formula of capreomycin sulfate is C_24_H_42_N_14_O_8_ × H_2_SO_4_ and the molecular weight is 752.8 g/mol).

(1) $$\begin{array}{c} Atomic\;{\%}_{nitrogen}\\ =\left[\frac{\left(M{\%}_{capreomycin\;sulfate}/m_{capreomycin\;sulfate}\right)\left(N_{capreomycin\;sulfate}\right)+\left(M{\%}_{mannitol}/m_{mannitol}\right)\left(N_{mannitol}\right)}{\left(M{\%}_{capreomycin\;sulfate}/m_{capreomycin\;sulfate}\right)\left(Z_{capreomycin\;sulfate}\right)+\left(M{\%}_{mannitol}/m_{mannitol}\right)\left(Z_{mannitol}\right)}\right]\left(100\right) \end{array}$$

where *M*% is the mass percentage of a compound in the formulation, *m* the molecular weight of the compound, *N* the number of nitrogen atoms in the compound, and *Z* the total number of atoms in the compound excluding hydrogen.

### 2.11. Animal Study

BALB/c mice of either gender aged 7 to 9 weeks weighing between 18 to 30 g were employed in this study. The mice were obtained from the Centre for Comparative Medicine Research, The University of Hong Kong. They were housed in a 12-h light/12-h dark cycle with food and water supplied ad libitum. All the animal experiments were performed with the approval from the Committee on the Use of Live Animals in Teaching and Research, The University of Hong Kong (CULATR 4921-19, approved on 1 February 2019).

### 2.12. Pharmacokinetic Study

The mice were randomly divided into two treatment groups (*n* = 45 per group, five mice per time point, nine time points in total). The intravenous (IV) group received capreomycin (sulfate) solution prepared in normal saline (4 mg/mL) via tail vein injection at a dose of 20 mg/kg [2,13]. Intratracheal (IT) group received the C_25__T90 formulation as powder aerosol intratracheally as previously described [25]. In brief, the powder was loaded in a 200 µL gel loading pipette tip, which was connected with a three-way stopcock and 1 mL syringe. The pipette tip was inserted into the trachea of mice under anesthesia. The powder was dispersed into the lungs of mice by 0.6 mL of air in the syringe. After each powder insufflation, the pipette tip was removed and rinsed with 1 mL ultra-pure water. The rinsing solution was assayed by HPLC to calculate the residual mass of capreomycin in the tip. The delivered mass of capreomycin was calculated as drug content in the loaded mass of powder minus the residual mass of capreomycin in the tip. The insufflation efficiency (%) was the ratio of delivered mass of capreomycin to the loaded mass of capreomycin. According to a preliminary study, the insufflation efficiency of C_25__T90 formulation was around 80% (unpublished data). To achieve a dose comparable with the IV group, 25 mg/kg of capreomycin was initially loaded and administered in the IT group (around 2 mg powder for a 20 g mouse). The delivered dose within 17.5 to 22.5 mg/kg was considered successful administration, and mice that received dose outside this range were excluded. At 5 min, 15 min, 30 min, 1 h, 2 h, 4 h, 6 h, 8 h, and 24 h post-administration, the mice were euthanized with overdose pentobarbital (200 mg/kg). The whole blood was collected by cardiac puncture, followed by centrifugation at 13,000 rpm for 10 min to obtain the plasma. The lungs were collected and frozen in liquid nitrogen immediately. The plasma and lung tissues were stored at −80 °C until further analysis.

### 2.13. Extraction of Capreomycin

The extraction of capreomycin from plasma and tissue homogenates was performed according to a previous study [2] with minor modification. Tissue homogenates were obtained by homogenizing the tissue with sterile water. A volume of 1 µL perchloric acid was added to a microcentrifuge tube containing 100 µL of plasma or tissue homogenates to induce protein precipitation. The mixtures were vortexed for 10 min and centrifuged at 16,000× *g* for 10 min. The supernatant was collected and neutralized with 110 µL basic mix of 1:10 KOH/K_2_HPO_4_ solution. The obtained mixtures were then vortexed, followed by centrifugation at 16,000× *g* for 15 min. The supernatant was transferred to glass vials for HPLC analysis. A standard curve was obtained by spiking known concentrations of capreomycin (0.6 to 40.0 µg/mL) into blank plasma or blank lung tissue homogenate followed by treatment as mentioned above. HPLC was performed using a C18 column (5 μm, 4.6 × 250 mm, Agilent, Santa Clara, CA, USA) with a guard cartridge. In this HPLC method, HFBA replaced TFA to improve peak resolution of the samples. The mobile phase consisted of 0.1% HFBA in ultrapure water (solvent A) and 0.1% HFBA in HPLC grade acetonitrile (solvent B), pumped at 1 mL/min under the following gradient: 0 to 2 min, 80% A; 2 to 22 min, 80–20% A; 22 to 25 min, 20% A (*v*/*v*). A volume of 50 µL sample was injected and capreomycin was detected by UV absorption at 268 nm. Capreomycin eluted at approximately 9.7 min with this method. Linearity was demonstrated between 0.6 and 40.0 mg/mL (*R*^2^ = 0.998).

### 2.14. Data Analysis

The FPF data evaluated by NGI was analyzed by one-way ANOVA followed by Tukey post hoc test by GraphPad Prism (version 8.0.1, GraphPad Software, San Diego, CA, USA). For the pharmacokinetics data, the maximum concentration of capreomycin (*C*_max_) and time to obtain the maximum concentration (*T*_max_) in plasma and lung tissues, the elimination rate constant (*K_el_*), half-life (*t*_1/2_), clearance (CL), area under the curve from 0 h to *t* (AUC_0–t_), area under the curve from 0 h to infinity (AUC_0–∞_) and mean residence time (*MRT*) in plasma and lung tissue were calculated by noncompartmental methods (Phoenix WinNonlin, Certara, Princeton, NJ, USA). Pharmacokinetic parameters were analyzed by Student’s *t*-test.

## 3. Results

### 3.1. Production Yield of Spray Drying

The production yield of spray drying was affected by both the capreomycin content in the formulation and the inlet temperature (Table 2). In general, as the capreomycin content increased, the production yield decreased. The inlet temperature of 60 °C (the lowest temperature employed in the study) resulted in the lowest production yield within the group of the same drug content. In the C_20_ and C_25_ groups, when the inlet temperature was 90 °C or above, formulations had relatively high yield of over 60%. Due to the low production yield of C_50_ group (all below 10%), the formulations in this group were not further investigated in the subsequent studies.

### 3.2. Drug Content and Residual Moisture

The measured drug content was close to the theoretical value in all tested formulations (Table 2). The residual moisture of spray-dried powders ranged from 0.48% to 3.13% (*w*/*w*). Low inlet temperature (60 °C) generally produced particles with higher moisture level than other formulations with the same capreomycin content. There was no clear trend between residual moisture and drug content.

### 3.3. Particle Morphology

**Figure 1:**
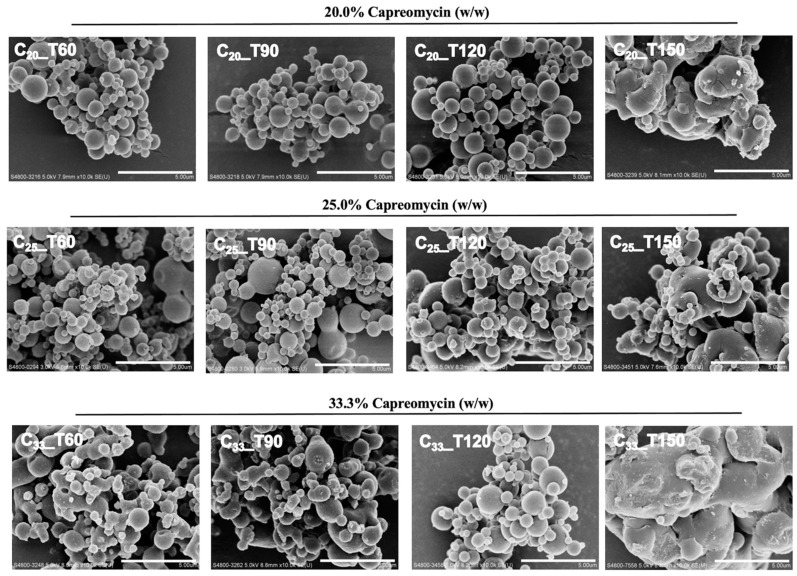
The scanning electron microscopy (SEM) images of spray-dried powder imaged at ×10,000 magnification, scale bar = 5 μm.

As observed in the SEM images (Figure 1), spray-dried particles prepared at inlet temperature of 120 °C or below were generally spherical in shape with smooth surface and partially aggregated. When the drug content increased from 20% to 33% (*w*/*w*), the particles appeared to be progressively fused together to produce an interconnected structure, which was particularly prominent in C_33__T60 and C_33__T90 formulations. As the inlet temperature further increased to 150 °C, the particles were no longer spherical and instead showed a rough surface with large and irregularly shapes.

### 3.4. Particle Size Distribution

The volumetric size distribution of spray-dried powder was measured by laser diffraction (Table 3). When the powder was prepared at a high inlet temperature of 150 °C, the particles were larger than those prepared at a lower inlet temperature, which was consistent with the SEM images. The T120 group exhibited the smallest volumetric diameter compared to those spray-dried with the same drug content at other inlet temperatures. Apart from T150 group, the higher inlet temperature, the smaller the particle size. In T60, T90 and T150 groups, when the content of capreomycin was increased to 33% (*w*/*w*), the particle size became larger. C_20__T120 showed the smallest D_50_ of 1.82 μm while C_33__T150 showed the largest D_50_ of 6.44 μm. Compared with other formulations, C_25__T150 and C_33__T150 showed a wider span (>2).

### 3.5. In Vitro Aerosol Performance

**Figure 2:**
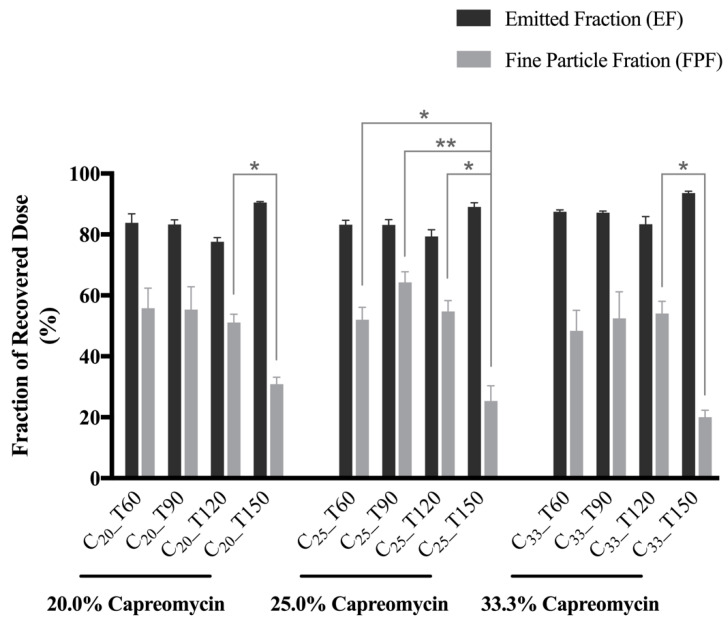
In vitro aerosol performance of spray-dried powders evaluated by the Next Generation Impactor (NGI). Data presented as mean ± standard deviation (*n* = 3). *p*-value indicated comparison of FPF between formulations with the same drug content (* and ** represents *p* < 0.05 and *p* < 0.01, respectively, one-way ANOVA followed by Tukey post hoc test).

The aerosol performance of the spray-dried powders from NGI experiments are presented as EF, FPF (Figure 2) and MMAD (Table 3). All the spray-dried powders showed similar dispersion trends with EF approaching or over 80%. In the C_20_ and C_33_ groups, the powders prepared at an inlet temperature of 150 °C showed significantly lower FPFs than those prepared at 120 °C (*p* < 0.05, one-way ANOVA by Tukey post hoc test). In the C_25_ group, the FPF of C_25__T150 was significantly lower than all other formulations with the same drug content (*p* < 0.05 or 0.01, one-way ANOVA by Tukey post hoc test). All the formulations prepared at 120 °C or below had FPF values of over 50%. The MMAD of particles in the T150 group were larger than that of other formulations, which was consistent with the SEM images and volumetric diameter. Formulations that were prepared at 120 °C or below showed similar MMAD within the range of 3.4 to 5.3 μm regardless of the formulation composition, indicating that the inlet temperature should not be higher than 120 °C in order to produce inhalable powder. Among all the formulations investigated, C_25__T90 demonstrated the highest FPF of over 64% with the smallest aerodynamic diameter of 3.38 µm. Therefore, it was identified as the optimal formulation for the pharmacokinetic study.

### 3.6. Thermoanalysis and Powder Crystallinity

**Figure 3:**
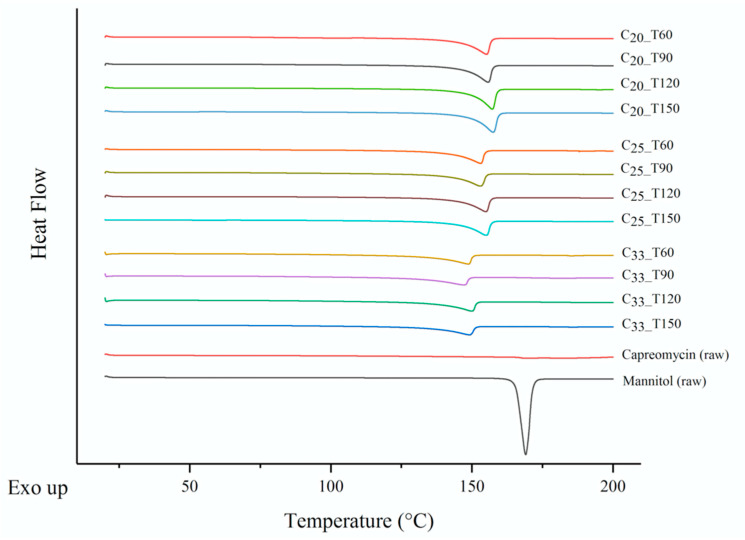
Differential scanning calorimetry (DSC) thermogram of spray-dried powders, raw capreomycin and raw mannitol. Negative peak represents endothermic events.

**Figure 4:**
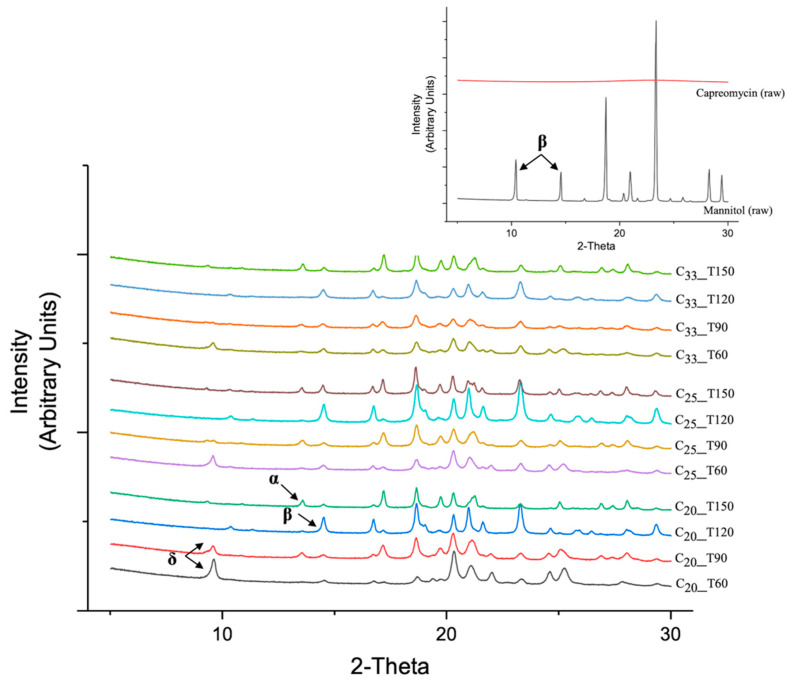
X-ray powder diffraction (PXRD) diffractogram of raw capreomycin, raw mannitol (right conner) and spray-dried powders (from 5° to 30°).

All investigated formulations demonstrated a characteristic endothermic peak at 150 to 160 °C in the DSC study (Figure 3), whereas raw mannitol was reported to have a melting point at 166 to 168 °C [26]. This observation demonstrated a shift in melting point of mannitol in the presence of capreomycin. The higher the content of capreomycin, the lower the temperature of the endothermic peak. Formulations with the same content of capreomycin showed similar thermal behavior regardless of the inlet temperature of spray drying. The smaller particles size of mannitol after spray drying may also cause melting point depression [27]. According to the PXRD results (Figure 4), raw mannitol was highly crystalline and predominately in the β form, as indicated by the peaks at 10.5° and 14.7°(indicated by arrows) [28]. Formulations in the T120 group were also predominately in the β form (14.7°, indicated by the arrow in C_20_ group) while those in the T150 group exhibited α and β polymorphs, since they had the characteristic peaks not only at 14.7° but also at 13.6° (indicated by the arrow in C_20_ group) [28]. Interestingly, compared to the diffractogram of pure δ forms of _D_-mannitol, the mannitol in T60 and T90 groups also contained the δ form (characteristic peak at 9.74° as the arrow indicated in C_20_ group) [28,29], which is thermodynamically less stable than the other two forms [29,30].

### 3.7. Surface Composition

**Figure 5:**
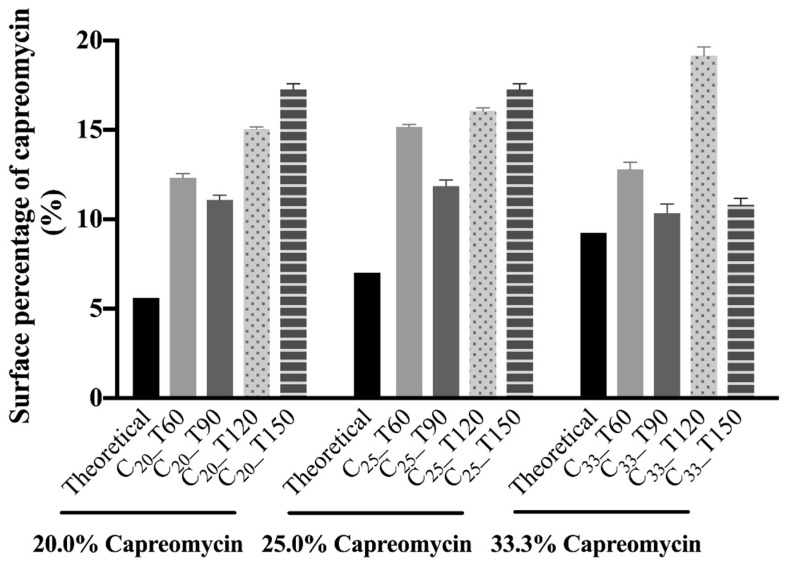
Surface percentage of capreomycin in different spray-dried powders.

The elemental composition of raw mannitol and capreomycin sulfate by the number of atoms in the molecules (Table 4), and the molar surface capreomycin sulfate composition of the spray-dried powders (Figure 5) were calculated. The experimentally measured fraction of carbon was higher than the theoretical one for both capreomycin sulfate and mannitol, while those of oxygen and nitrogen (in capreomycin sulfate only) were lower. The measured surface proportion of sulfur in capreomycin sulfate was close to the theoretical value. The theoretical molar proportion of capreomycin sulfate in C_20_, C_25_ and C_33_ groups were 5.6%, 7.0%, and 9.3%, respectively. All spray-dried formulations displayed an enrichment of capreomycin on the surface of the particles. Among the formulations of the same drug content, those spray-dried at 90 °C had the lowest capreomycin sulfate surface coverage. Only formulations spray-dried at 120 °C showed a clear trend of increasing capreomycin sulfate surface coverage with the drug load, with C_33__T120 exhibiting the highest content of capreomycin sulfate on the surface (19.2% *w*/*w*). No clear trend was observed for other inlet temperatures.

### 3.8. Pharmacokinetic Study

**Figure 6:**
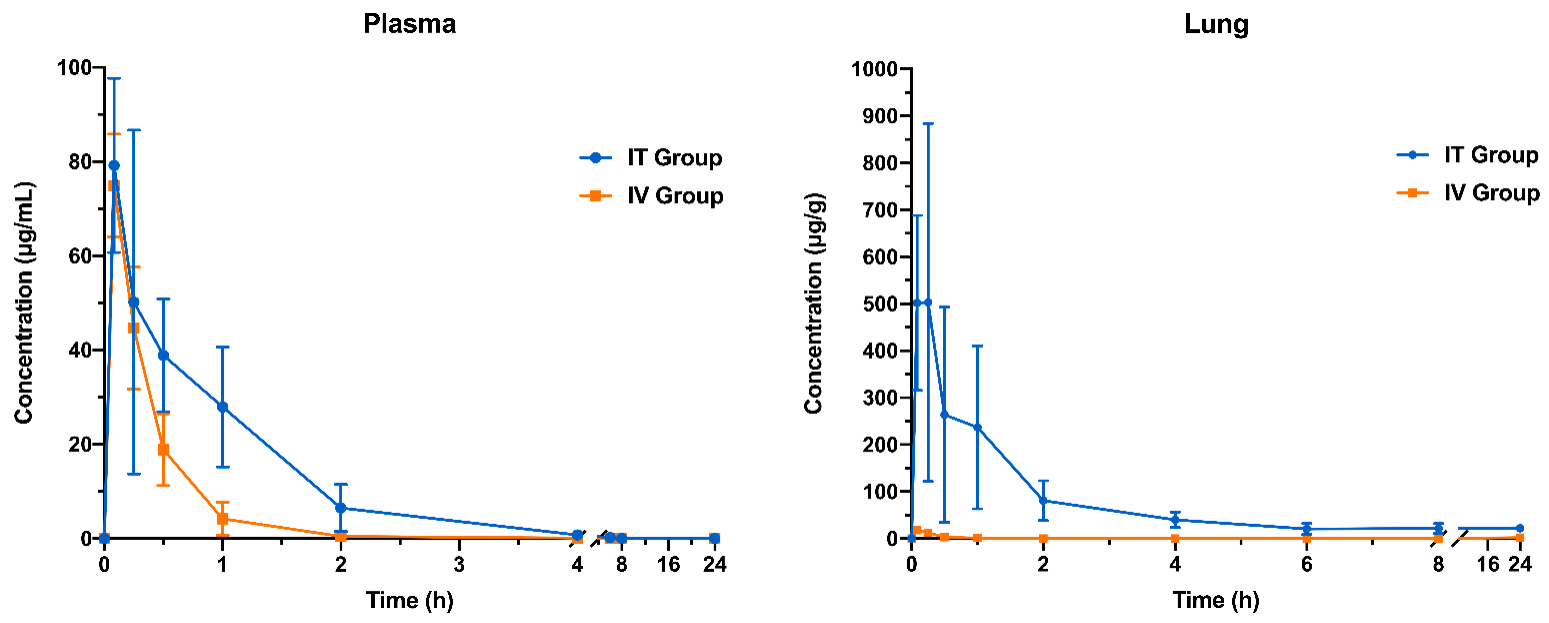
Pharmacokinetic study of spray-dried powder formulation of capreomycin on mice. The mice in intratracheal (IT) group were administered with capreomycin spray-dried powders (C_25__T90 formulation) intratracheally with a dosage range of 17.5–22.5 mg/kg (the average delivered dose was 20.54 ± 1.31 mg/kg). The mice in intravenous (IV) group were administered with capreomycin solution intravenously at a dose of 20 mg/kg. Data was presented as mean ± standard deviation. (*n* = 5 for each time point).

The pharmacokinetic profiles of capreomycin following IT and IV administration (as C_25__T90 powder and capreomycin solution, respectively) were compared (Figure 6) and the pharmacokinetics parameters in plasma and lung tissue were calculated by noncompartmental analysis (Table 5). In the plasma, the *C*_max_ was comparable between IT and IV group, approaching 80 µg/mL. Both groups achieved maximum capreomycin concentration within 10 min (*T*_max_) after administration. However, the concentration of capreomycin declined at a slower rate in the IT group than the IV group until it was below the detection limit at 4 h. Although the *K_el_* and *t*_1/2_ of the two groups did not show any significant difference, the CL of the IT group was significantly lower than that of the IV group (*p* < 0.001 Student’s *t*-test), and accordingly, the MRT in the IT group was significantly longer than in the IV group (*p* < 0.01 Student’s *t*-test). The AUC_0–t_ and AUC_0–__∞_ in IT group were around two-fold higher than that in the IV group (*p* < 0.01, Student’s *t*-test). In the lung tissues, both IT and IV group achieved *C*_max_ within 20 min after administration. However, the *C*_max_ in the IT group was 40-fold higher than that in IV group (*p* < 0.001). The capreomycin concentration in the lung tissue of the IT group declined very slowly (significantly smaller *K_el_* compared to the IT group, *p* < 0.001, Student’s *t*-test) and the drug could still be detected after 24 h of administration. In contrast, capreomycin was no longer detectable in the lung 1 h after IV administration, with a significantly shorter MRT in the lung compared to the IT group (*p* < 0.001, Student’s *t*-test). The CL in the lung tissue of IT group was significantly lower than that of the IV group (*p* < 0.001), while both AUC_0–t_ and AUC_0–__∞_ of the IT group were 150-fold (*p* < 0.01, Student’s *t*-test) and 220-fold (*p* < 0.001, Student’s *t*-test) higher than that of the IV group, respectively.

## 4. Discussion

Capreomycin is the first choice of the second-line anti-TB drugs for the treatment of MDR-TB. It is administered intramuscularly or intravenously up to 1.0 g per day, five times per week [16,31]. The high systemic exposure of capreomycin has led to nephrotoxicity and ototoxic effects in some patients [32,33]. To overcome the main challenge of toxicity and inconvenient administration, pulmonary drug delivery is an attractive administration route because it provides high drug concentration in the lungs and therefore decreases the dose required [31]. Inhaled therapy can reduce the risk of systemic adverse effects by targeting the drug directly to the lesion where *Mtb* typically reside. Despite the advantages of inhalation therapy against TB, there is no commercial inhalable product approved for TB. Spray drying is one of the most popular particle engineering technologies investigated for preparing inhalable dry powder of antibiotics, including antimicrobial peptides, for inhaled TB therapy [34,35,36]. It is a continuous process that is easy for scale-up and its closed system facilitates aseptic industrial production. Furthermore, spray drying has the advantage of having good control of particle size distribution, which is critical for inhaled formulations [37]. However, spray-dried powders tend to have low crystallinity, which often leads to particle aggregation and moisture absorption, rendering it unfavorable for long term storage [38].

Among the anti-TB drugs being investigated for inhalation, only one clinical study involved inhalable powders of capreomycin, in which safety, tolerability, and pharmacokinetic profile of a spray-dried formulation of capreomycin (containing drug to l-leucine at a mass ratio of 80:20) were evaluated in healthy subjects [14]. The inhaled formulation of capreomycin was well-tolerated, indicating that inhaled therapy of capreomycin is feasible. However, the production process involved the use of organic solvent (50% ethanol), and the hydrophobic l-leucine has dissolution issue. The use of organic solvent in the production process may increase the cost of manufacture with an increased risk of irritation to the pulmonary mucosa due to the residual solvent. Schoubbe et al. prepared capreomycin powder formulation with a nano-spray dryer [15]. According to the aerodynamic assessment evaluated by the twin-stage glass impinger with the Handihaler^®^ at the flow rate of 60 L/min, the addition of lactose improved the respirable fraction of spray-dried capreomycin from 14% to 26%, but the large proportion of lactose in the formulation (capreomycin to lactose at 1:50 mass ratio) has limited the respirable dose of capreomycin. Compared with lactose, mannitol is a non-reducing sugar and less hygroscopic. Therefore it shows better compatibility and stability [39]. The safety of mannitol as inhalation excipient was well examined, and it has been used in commercial pharmaceutical protein formulations such as Exubera^®^, an inhaled insulin product approved by Food and Drug Administration (FDA) [39,40]. In addition, inhaled mannitol was also approved by the FDA as the active pharmaceutical ingredient (Bronchitol^®^) for the management of cystic fibrosis. This study aimed to develop a new inhaled powder formulation of capreomycin with mannitol by spray drying. Different drug contents and inlet temperatures during spray drying were investigated to identify the optimal formulation and spray drying conditions to produce capreomycin powders suitable for inhalation.

Both the drug content in the formulation and inlet temperature of spray drying can influence the production yield. When the temperature was low, drying was incomplete, leading to the adherence of droplets on the inner surfaces of the cyclone and hence low production yield [41,42,43]. In the C_20_, C_25_ and C_33_ groups, the production yield was the highest at the inlet temperature of 120 °C, above which the yield started to decline. In the C_50_ group, there was a high content of capreomycin, which is amorphous in nature with high surface adhesive property [44]. As a result, the particles, in general, were more likely to adhere to the cyclone of the spray dryer instead of depositing in the collector vial. The inlet temperature also influenced the residual moisture in the spray-dried powder. As expected, spray drying at low temperature (inlet temperature of 60 °C) produced particles with higher residual moisture than other groups. No clear trend was observed in the residual moisture between T90, T120, and T150 groups. The result suggested that an inlet temperature of 60 °C was insufficient for proper drying, which led to the low production yield and high residual moisture.

The morphology of particles produced by spray drying was mainly affected by temperature rather than the capreomycin content. Unlike the T60, T90 and T120 formulations, particles of the T150 group were irregular in shape with rough surfaces. This can be explained by the different crystallization process of mannitol at different temperatures [45,46]. When the temperature reached a certain level (e.g., at 150 °C in this study), solvent evaporated very rapidly. The high solvent evaporation rate with a low crystallization nucleation rate rendered the feed liquid to become highly concentrated and viscous. As a result, larger mannitol crystals with rough surfaces were formed [45,46]. In contrast, the particles of T60, T90 and T120 groups were spherical, which have a small area-to-volume ratio that facilitates powder dispersion by reducing aggregation [47]. The particle size distribution was a decisive factor of the deposition site following inhalation. It is widely accepted that particles with median aerodynamic diameters in the range of 1 to 5 μm can efficiently deposit in the lower airways [48]. Formulation C_25__T90 had the smallest MMAD of 3.38 μm, indicating that this formulation is likely to achieve efficient lung deposition. The similar particle size between T60, T90 and T120 groups can be explained by the particle formation process [45,49,50]. During the drying process at lower inlet temperature (at 120 °C and below), the water evaporation rate was low, and the mannitol began to crystallize once it reached a critical concentration at the surface [45,49,50]. Therefore, the particles size was mainly determined by the droplet size. As the droplet size after atomization was similar between formulations with the same composition and spray drying parameters except for inlet temperature (which was not high enough to make any difference), the similar drying manner may produce particles with similar size [45,49,50]. In contrast, when the inlet temperature was high enough (at 150 °C or above), the solvent evaporated very rapidly, and the crystallization growth may be different due to supersaturation. The mannitol tended to crystallize with a secondary nucleation process, for instance, using other already crystalline particles in the spray dryer as seed, leading to a wide variation of particle size [45]. The particles in T150 groups with rough surface may also have different particle interaction compared to the smooth particles [45], hence affecting the resultant particle size.

Crystallinity plays an important role in the stability of powder formulation. For pure mannitol, the crystalline form was mainly determined by solvent evaporation rate during spray drying [45]. According to hot stage microscopy experiments in another study, when mannitol was heated to around 90 °C, the β form was dominant (95%); when mannitol was heated to a higher temperature of around 140 °C, a mixture of polymorphs containing both β form (85%) and α form (15%) were observed [45]. This phenomenon was consistent to our T120 and T150 groups. In the presence of capreomycin, the δ form of mannitol, which is the least stable crystalline form [51], was found in the formulations of T60 group and T90 group. Nonetheless, the mannitol in all spray-dried powders was crystalline, suggesting that they have good stability for long-term storage [52], but further investigation is needed.

In all spray-dried formulations, an enrichment of capreomycin at the particle surface was observed, which can be explained by the dimensionless Péclet number (*Pe*; Equation (2)):

(2) $$Pe=\frac{\kappa }{8D_{j}}$$

where *D_j_* is the solute diffusivity and *κ* is the evaporation rate [53,54]. A large *Pe* indicates a higher droplet surface recession rate than solute diffusion inwards during droplet evaporation, leading to surface enrichment by the molecule with slower diffusion rate [55]. At a given evaporation rate, the *Pe* of capreomycin is higher than that of mannitol due to its lower *D_j_*, owing to its higher molecular weight and lower aqueous solubility, suggesting that capreomycin precipitated first and predominantly occupied at the surface. The XPS data were consistent with this phenomenon as more capreomycin was detected on the particle surface than expected in all spray-dried formulations. According to Equation (2), *Pe* increases with the evaporation rate, which can be achieved by increasing the inlet temperature of spray drying and changing the mass ratio of the components [56]. Therefore, drug surface coverage is expected to increase with the inlet temperature and drug load. However, the capreomycin sulfate surface coverage did not exactly follow this trend. The capreomycin sulfate surface coverage increased with drug loading in the formulation only at 120 °C but varied at other temperatures. The effects of drug loading, temperature, and their interaction on surface coverage have not been studied systematically. Mangal et al. studied the spray-dried polyvinylpyrrolidone with different concentrations of l-leucine (0, 2.5, 5, 7.5, 10, 12.5, and 15% *w*/*w*) [57]. Their XPS results showed that the surface coverage by l-leucine increased with its concentration and reached a plateau at 12.5% *w*/*w*. However, this observation may be formulation dependent. More research is required to establish the relationship between spray drying conditions, drug loading, and surface coverage.

Among all the formulations prepared in this study, C_25__T90 demonstrated the best aerosol properties in terms of FPF and MMAD with a reasonably good production yield. This formulation was selected for subsequent pharmacokinetic study in animals. The inhaled formulation of capreomycin was designed for high local concentration in the lung with reduced systemic adverse effects. The minimum inhibitory concentration (MIC) of capreomycin in liquid or on solid media is 1.25 to 2.5 µg/mL [58]. In the pharmacokinetic study, when administrated through pulmonary delivery, the capreomycin concentration in the lungs of mice were higher than the MIC and remained stable at around 20 µg/g from 6 to 24 h post-administration (8-fold higher than the MIC). As the lung is the primary site of *Mtb* infection, maintaining capreomycin concentration within the therapeutic window for an extended period in the lung can combat the bacteria more effectively [14]. Drug resistance can develop when the intracellular drug concentration does not reach the microbiologically active level during TB treatment [59]. Inhaled drug particles deposited in the deep lung would be ingested and phagocytosed by the alveolar macrophages and dendritic cells where the *Mtb* typically reside [8,60], resulting in high drug concentration in the infected cells. In addition, these drug-containing alveolar macrophages may migrate to the periphery of lung granulomas and facilitates the delivery of drug to the mycobacteria inside granulomas, where it was hard for the drug to achieve therapeutic concentration via circulation through injection [59]. According to the pharmacokinetics and pharmacodynamics studies of inhaled capreomycin particles in guinea pig model [2,12], animals receiving capreomycin by inhalation showed significantly higher drug concentration in the lungs and bronchoalveolar lavage fluid than intramuscular injection. Moreover, insufflation with high dose of capreomycin (14.5 mg/kg capreomycin) could lighten the bacterial burdens (CFU/mL) in the lungs more efficiently compared to intramuscular injection. High local drug concentration not only can reduce bacteria load in the lung, but more importantly, minimize the possibility of airborne transmission of bacteria to other individuals [59].

The inhaled formulation of capreomycin can achieve comparable drug concentration to capreomycin solution administrated by IV injection in the plasma through alveolar-capillary absorption, indicating that it can also treat extrapulmonary TB infection [14]. Capreomycin is associated with nephrotoxicity and ototoxicity, similar to the aminoglycosides [33,61]. The higher AUC in both the plasma and the lung obtained from the mice in IT group compared to IV group suggested that inhaled formulation of capreomycin showed higher bioavailability. To achieve comparable drug efficacy, a lower dose can be used, leading to less systemic exposure, and reduced adverse effects. The slower clearance and longer mean residence time in the mice of IT group indicated that capreomycin received by inhalation eliminated slower than by intravenous injection, which may help to reduce the dosing frequency. With the favorable aerosol properties and pharmacokinetic profile of C_25__T90 formulation, further study on its antibacterial effect in vivo is warranted.

## 5. Conclusions

Powder formulations containing different mass ratios of capreomycin to mannitol were prepared by spray drying at different inlet temperatures. Both factors influenced the production yield and residual moisture of the spray-dried powder. To achieve a good production yield, the formulation should have capreomycin content below 50% (*w*/*w*) and spray-dried at an inlet temperature of 90 °C or above. Except for the T150 groups, all spray-dried formulations in this study displayed spherical morphology with smooth surface and similar particle size distribution. Among all the formulations, C_25__T90 exhibited the best aerosol performance with the MMAD of 3.38 μm and FPF approaching 65%. This formulation not only showed better aerosol properties than the other two reported dry powder formulations of capreomycin but also with simpler and organic solvent-free preparation process. The pharmacokinetics study on healthy mice indicated that higher maximum concentration was achieved in the lung of mice receiving inhaled powder formulation than those receiving capreomycin solution by intravenous injection. Up to 24 h post-administration of the inhaled powder formulation, the drug concentration in the lungs was 8-fold higher than the MIC. Inhaled capreomycin formulations also showed slower clearance in the plasma than IV injection. While long-term stability and pharmacodynamic study remains to be investigated, this spray-dried formulation of capreomycin is promising for use in inhaled TB therapy.

## Figures and Tables

**Table 1:** The composition of spray-dried powders prepared at different inlet temperature.

Sample Name	Capreomycin: Mannitol Ratio (*w*/*w*)	Capreomycin Percentage by Mass	Inlet Temperature (°C)
C_20__T60	1:4	20.0	60
C_20__T90			90
C_20__T120			120
C_20__T150			150
C_25__T60	1:3	25.0	60
C_25__T90			90
C_25__T120			120
C_25__T150			150
C_33__T60	1:2	33.3	60
C_33__T90			90
C_33__T120			120
C_33__T150			150
C_50__T60	1:1	50.0	60
C_50__T90			90
C_50__T120			120
C_50__T150			150

**Table 2:** The outlet temperature, production yield, drug content and residual moisture of spray-dried powders. Data for drug content was presented as mean ± standard deviation (*n* = 3). N.A. Not applicable. Due to the low production yield of C_50_ group, the spray-dried powders of this group were not further investigated.

Sample Name	Outlet Temperature (°C)	Production Yield (%, *w*/*w*)	Drug Content (%, *w*/*w*)	Residual Moisture (%, *w*/*w*)
C_20__T60	36–38	53.0	19.2 ± 0.4	2.2
C_20__T90	52–55	69.9	18.7 ± 1.4	1.2
C_20__T120	70–74	66.9	19.8 ± 0.3	1.6
C_20__T150	90–95	63.3	19.0 ± 0.6	1.4
C_25__T60	35–39	47.3	25.5 ± 0.3	2.3
C_25__T90	52–56	65.7	24.9 ± 0.6	0.7
C_25__T120	68–71	78.1	26.7 ± 0.4	1.5
C_25__T150	83–86	63.9	24.5 ± 0.8	0.5
C_33__T60	34–37	18.2	33.2 ± 0.6	3.1
C_33__T90	53–56	25.8	33.6 ± 0.4	2.8
C_33__T120	68–70	52.5	34.4 ± 0.8	2.0
C_33__T150	88–92	39.1	34.1 ± 0.2	2.8
C_50__T60	34–37	1.0	N.A.	N.A.
C_50__T90	52–57	6.9	N.A.	N.A.
C_50__T120	66–68	4.3	N.A.	N.A.
C_50__T150	84–87	7.2	N.A.	N.A.

**Table 3:** Particle size distribution of spray-dried powders. The volumetric diameter was obtained from laser diffraction measurement (Flow rate: 60 L/min, inhaler: Breezhaler, capsule: gelatin). The median aerodynamic diameter (MMAD) and geometric standard deviation (GSD) was obtained from aerosol performance analysis (Next generation impactor, flow rate: 90 L/min, duration time: 2.7 s, inhaler: Breezhaler, capsule: gelatin). Data for volumetric diameter and aerodynamic size was presented as mean ± standard deviation (*n* = 3).

Sample	Volumetric Diameter				Aerodynamic Diameter	
	D_10_ (μm)	D_50_ (μm)	D_90_ (μm)	Span Value	MMAD (μm)	GSD
C_20__T60	1.21 ± 0.02	2.67 ± 0.02	5.20 ± 0.14	1.49 ± 0.03	4.29 ± 0.77	3.09 ± 0.14
C_20__T90	1.20 ± 0.02	2.52 ± 0.00	4.59 ± 0.10	1.35 ± 0.05	4.28 ± 0.85	3.30 ± 0.28
C_20__T120	0.76 ± 0.03	1.82 ± 0.06	3.47 ± 0.04	1.49 ± 0.07	4.58 ± 0.37	3.89 ± 0.33
C_20__T150	1.66 ± 0.04	5.36 ± 0.17	10.67 ± 0.44	1.68 ± 0.03	9.85 ± 1.15	4.02 ± 0.63
C_25__T60	1.32 ± 0.08	3.07 ± 0.09	6.51 ± 0.37	1.69 ± 0.07	4.46 ± 0.21	2.45 ± 0.25
C_25__T90	1.16 ± 0.01	2.62 ± 0.05	5.06 ± 0.16	1.49 ± 0.03	3.38 ± 0.22	2.83 ± 0.14
C_25__T120	0.97 ± 0.03	2.44 ± 0.04	4.80 ± 0.06	1.57 ± 0.03	4.28 ± 0.36	2.70 ± 0.25
C_25__T150	1.28 ± 0.04	5.16 ± 0.14	11.73 ± 0.22	2.03 ± 0.03	8.79 ± 0.38	2.72 ± 0.20
C_33__T60	1.57 ± 0.03	3.33 ± 0.02	6.83 ± 0.15	1.58 ± 0.05	5.29 ± 0.93	3.06 ± 0.06
C_33__T90	1.49 ± 0.05	3.16 ± 0.05	6.51 ± 0.24	1.59 ± 0.05	4.74 ± 1.14	3.08 ± 0.26
C_33__T120	1.08 ± 0.05	2.60 ± 0.11	5.17 ± 0.37	1.57 ± 0.07	4.32 ± 0.34	2.35 ± 0.31
C_33__T150	2.16 ± 0.21	6.44 ± 0.54	15.74 ± 2.83	2.10 ± 0.25	16.59 ± 2.66	3.87 ± 0.31

**Table 4:** Theoretical and experimental percentages by the number of atoms of elements in pure capreomycin (as sulfate) and mannitol.

Element	Raw Capreomycin (as Sulfate)		Raw Mannitol	
	Theoretical	Experimental	Theoretical	Experimental
Carbon	47.1	57.2 ± 0.2	50.0	52.7 ± 0.2
Oxygen	23.5	19.0 ± 0.1	50.0	47.3 ± 0.2
Nitrogen	27.5	21.7 ± 0.1	-	-
Sulphur	2.0	2.1 ± 0.0	-	-

**Table 5:** Pharmacokinetic parameters obtained by noncompartmental analysis after administration of capreomycin by pulmonary or intravenous route. The mice in intratracheal (IT) group were administered with capreomycin spray-dried powders (C_25__T90 formulation) intratracheally with a dosage range of 17.5–22.5 mg/kg. The mice in intravenous (IV) group were administered with capreomycin solution intravenously at a dose of 20 mg/kg. Data was presented as mean ± standard deviation (*n* = 5).

Parameters ^a^	Plasma		Lung	
	IT Group	IV Group	IT Group	IV Group
*K_el_* (h^−1^)	1.15 ± 0.57	2.74 ± 1.67	0.07 ± 0.04 ***	2.37 ± 0.23 ^b^
*t*_1/2_ (h)	0.73 ± 0.33	0.34 ± 0.20	18.94 ± 20.67	0.29 ± 0.03 ^b^
CL (mL/h·kg)	321.88 ± 81.65 ***	612.91 ± 93.46	12.99 ± 3.66 ***	2624.71 ± 157.19 ^b^
AUC_0–t_ (µg·h/mL)	65.10 ± 16.37 **	32.55 ± 5.52	1061.88 ± 235.76 ***	6.71 ± 0.51
AUC_0–∞_ (µg·h/mL)	67.26 ± 16.92 **	33.78 ± 5.54	1726.37 ± 658.83 **	7.78 ± 0.46 ^b^
MRT (h)	0.79 ± 0.22 **	0.30 ± 0.11	6.62 ± 1.25 ***	0.23 ± 0.13
*C_max_* (µg/mL) or (µg/g) ^c^	80.08 ± 18.84	74.89 ± 10.94	739.13 ± 180.66 ***	18.23 ± 8.34
*T_max_* (h)	0.15 ± 0.09	0.08 ± 0	0.27 ± 0.20	0.12 ± 0.07

^a^*K_el_*, elimination rate constant; *t*_1/2_, half-life; CL, clearance; AUC_0–t_, area under the curve from 0 h to *t*; AUC_0–∞_, area under the curve from 0 h to infinity; MRT, mean residence time; *C_max_*, maximum concentration; *T_max_*, time at which *C_max_* occurs. ^b^ In the lungs of mice in the IV group, the drug concentration fell below the detection limit quickly. Each mouse was allocated to one of the five groups in each time point according to their body weight. Two of the five mice showed drug concentration below the detection limit at 30 min post-administration, hence two sets of data did not have enough points to fit the linear model for the calculation of the predicted parameters by WinNonlin. Only three sets of data were presented. ^c^ The unit of *C_max_* in the plasma was µg/mL and the unit of *C_max_* in the lung tissue was µg/g. ** or *** Significant difference between IT group and IV group (*p* < 0.01 or *p* < 0.001, Student’s *t*-test).

## Data Availability

Data are available upon request.
